# Interdisciplinary Research on Medieval Fresco Subjected to Degradation Processes in the Corbii de Piatră Cave Church

**DOI:** 10.3390/ma16155257

**Published:** 2023-07-26

**Authors:** Adriana Elena Vâlcea, Izabela Mariș, Aurelian Denis Negrea, Nicanor Cimpoeșu, Gheorghe Gârbea, Dorin Grecu, Sorin Georgian Moga, Bogdan Istrate, Flavio Nicolae Finta, Alin Daniel Rizea, Daniel-Constantin Anghel, Corneliu Munteanu, Mircea Ionuț Petrescu, Mărioara Abrudeanu

**Affiliations:** 1 Interdisciplinary Doctoral School, University of Pitesti, Str. Targu din Vale Nr. 1, 110040 Pitesti, Romania; adriana.valcea31@gmail.com (A.E.V.); fintaflavio@gmail.com (F.N.F.); 2 Department of Mineralogy, Faculty of Geology and Geophysics, University of Bucharest, 1 N. Bălcescu Ave., 011401 Bucharest, Romania; izabela@contentlogic.ro; 3 Regional Center of Research & Development for Materials, Processes and Innovative Products Dedicated to the Automotive Industry (CRC&D-AUTO), University of Pitesti, Str. Targu din Vale Nr. 1, 110040 Pitesti, Romania; denis.negrea@upit.ro (A.D.N.); sorin.moga@upit.ro (S.G.M.); 4 Department of Materials Science, Gheorghe Asachi Technical University of Iași, Bd. Dimitrie Mangeron, nr. 67, 700050 Iaşi, Romania; nicanor.cimpoesu@tuiasi.ro; 5 Faculty of Theology, Letters, History and Arts, University of Pitesti, Str. Targu din Vale Nr. 1, 110040 Pitesti, Romania; garbea_59@yahoo.com (G.G.); dorin.grecu@upit.ro (D.G.); 6 Mecatronics and Robotics Department, Faculty of Mechanical Engineering—Mechanical Engineering, Gheorghe Asachi Technical University of Iași, Bd. Dimitrie Mangeron, nr. 67, 700050 Iaşi, Romania; bogdan.istrate@academic.tuiasi.ro; 7 Department of Manufacturing and Industrial Management, University of Pitesti, 1 Târgul din Vale Street, 110040 Pitesti, Romania; alin.rizea@upit.ro (A.D.R.); daniel.anghel@upit.ro (D.-C.A.); 8 Technical Sciences Academy of Romania, Calea Victoriei, nr. 118, Sector 1, 010093 Bucuresti, Romania; 9 Department of Engineering and Management of Metallic Materials Casting, Politehnica University of Bucarest, Splaiul Independentei, 313, Sector 6, 060042 Bucureşti, Romania; ipetrescu@yahoo.com

**Keywords:** fresco, meteoric water infiltration, mortar, degradation, microstructure, chemical composition, adhesion

## Abstract

This paper presents research on the degradation processes of the fresco painting in the cave church of Corbii de Piatră Hermitage under the influence of meteoric infiltration water and environmental factors. The medieval fresco dates from the end of the 13th century and the beginning of the 14th century, being painted on a sandstone wall. The infiltration of meteoric water through this wall, the temperature variations, the environment and the repeated wetting/drying processes determined the degradation of the fresco, resulting in its detachment from large surfaces. This research established correlations between the processes that take place, the structural transformations, the changes in composition and the adhesion of the fresco to the sandstone wall. The results have been made available to conservation and restoration specialists, in order to choose appropriate materials and technologies. This paper presents findings regarding the pictorial material and introduces new analysis techniques in research on the degradation processes of the fresco painting in the cave church of Corbii de Piatră Hermitage under the influence of meteoric infiltration water and environmental factors.

## 1. Introduction

A unique medieval monument in the territory of Romania, the Corbii de Piatră cave church was built at the end of the 13th and the beginning of the 14th century on an old Dacian site, under the rule of Basarab I, the founder of Wallachia (1310–1352), with his votive painting being placed in the church {1,2,3}.

The Village Corbi had its first charter attestation from the royal chancery of Vladislav II on the 15th of April 1456, which confirms the authority of Master Mogos over several villages, which also included “Corbii de Piatră”. During the rule of Neagoe Basarab, two documents dated the 23rd of June and 27th of September attest that sister Magdalina is as the founder of Corbii de Piatră monastery. This was because she rebuilt it, as it was abandoned at the time. The documents attest that the monastery was dowered with many beneficences, and then rendered to Neagoe Basarab, so that “the monastery would be a princely one” [1]. The church is unique in Romania due to its antiquity, the purely Byzantine style of the fresco painting and the fact that it has two altars in the same nave.

The architecture of the Corbii de Piatră cave church is derived typologically from the hall churches, with two altars dedicated to a double rite, characteristic of the Byzantine world from the 10th century [1,2,3,4,5].

**Figure 1:**
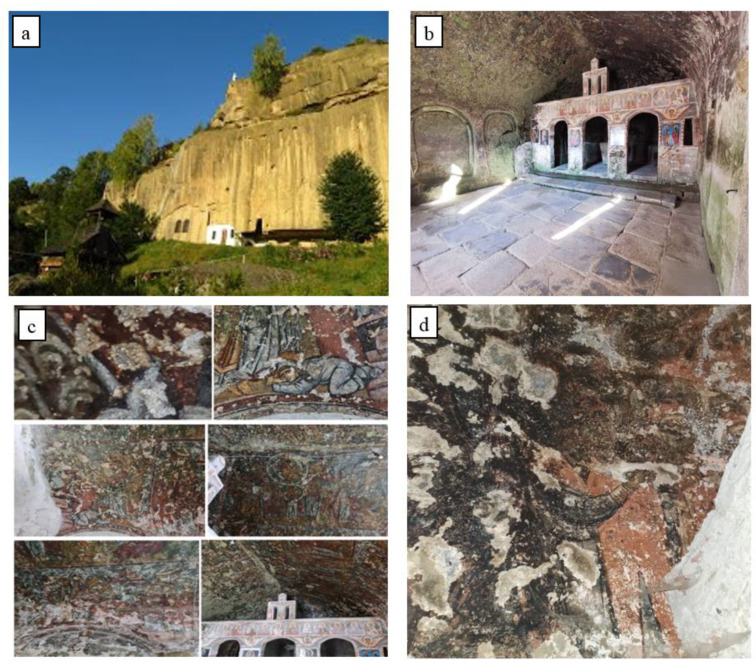
Corbii de Piatra cave church: (**a**) exterior view, tiled wall; (**b**) interior view [3,4,5]; (**c**) layout of the paintings in 2019; (**d**) sampling area (2022).

The al fresco painting was made at the beginning of the 14th century. Since the enclosure was excavated in the sandstone wall (Figure 1), the support on which the painting is applied has a relatively a uniform appearance, the roughness required for the application of the mortar layers for the fresco being provided by carving the sandstone. The support layers of the fresco are made on a lime base with straw inserts. Water infiltrations through the sandstone wall, temperature variations and biodegradation processes determined the advanced deterioration of the frescoes (Figure 1b–d).

Using advanced characterization techniques, the work shows the influence of aggressive factors on the structure, composition and adhesion of the fresco to the sandstone wall.

Taking into account the importance of the Corbii de Piatra Cave Church for the cultural patrimony and the fact that the degradation process has advanced rapidly during the last period, urgent measures should be taken in order to preserve and restore the church fresco. This paper presents a characterization of a fragment from the church fresco, strongly affected by the cumulative action of meteoric water infiltration and other aggressive factors. The research has been carried out by an interdisciplinary team and aims to provide scientific data about the original materials for art restorers. Research outcomes are also important from a historical point of view, with reference to the technique and materials used at the end of the 13th century in fresco paintings in Romanian territory.

## 2. Materials and Methods

The fragment of the fresco under investigation was taken from the southern wall of the church and is covered with a dark colored deposition layer (Figure 1d). The element of the fresco was taken from the southern wall of the church, at a height of 1.70 m from floor level, with the relevant area having a friable mortar, with a tendency to detach. The deontology of the conservation and restoration of historical monuments was respected during the sampling [6,7]. A single sample was taken, with a minimum volume, which was used for all the analyses performed. The characterization of the fresco fragment was carried out by: optical microscopy [8,9,10,11,12,13,14]; cathodoluminescence [13,15,16,17,18,19,20]; atomic force microscopy [21,22]; scanning electron microscopy with an energy dispersive fluorescence spectroscopy mode for elemental chemical analysis [8,9,10,11,12,23,24]; and X-ray diffraction [12,25,26,27,28,29].

The optical microscopy characterizations were performed in polarized light with a Zeiss Observer A1m optical microscope, which allows magnification up to 1000×, visualization in bright field, dark field, polarized light and acquisition of images with a Canon camera.

The microscopic analysis with cathodoluminescence was performed with Nikon E400, an optical microscope equipped with a cathodoluminescence device with a cold cathode (CL 8200 MK 3A). The images were acquired with a COOLPIX 950 digital photomicrograph device. The parameters required to perform the technique were the average vacuum value: 0.5 Torr; current voltage on the bundle: 15–17 kV; current intensity on the electron gun: 350–400 mA (according to the standard in use). The interpretation of cathodoluminescence results is qualitative.

Atomic force microscopy (AFM) was carried out with EasyScan II Nanosurf equipment using a non-contact mode, a CTR10 tip and Nanosurf software was used to evaluate the scans to use the fresco sample embedded in the resin for flatness, with the surface mechanically polished at a very low speed without removing the external paint layer. The experiments followed the determination of the surface layer morphology of the material constituents and the analysis of the intimate contact between them at their interface.

The characterization of the fresco element by SEM-EDS was carried out using the HITA-CHI SU5000 electron microscope, equipped with a backscattered electron detector and the energy dispersive fluorescence spectroscopy module for elemental analysis.

The X-ray diffraction analysis (XRD) was used to complete the identification of the crystal-line phases of the mortar components, and the painting layer X-ray diffractions (XRD) were performed using an XPERT PRO MPD 3060 facility from Panalytical (Alme-lo, The Netherlands), with a Cu X-ray tube (Kα = 0.154051 nm); 2 Theta: 20°–70°; step size: 0.13°; time/step: 51 s; and a scan speed of 0.065651°/s. The qualitative phase analysis was carried out using the PDXL2 program (Rigaku) and the PDF4+ 2022 database (International Center for Diffraction Data).

### 2.1. Optical Microscopy

Optical microscopy is frequently used for the characterization of heritage materials and goods [8,9]. The morphology, size and type of aggregates, inserts, compaction defects and interfaces were analyzed. For microscopic characterization, the fresco sample was embedded in resin and sanded on abrasive paper up to a roughness of 2000.

**Figure 2:**
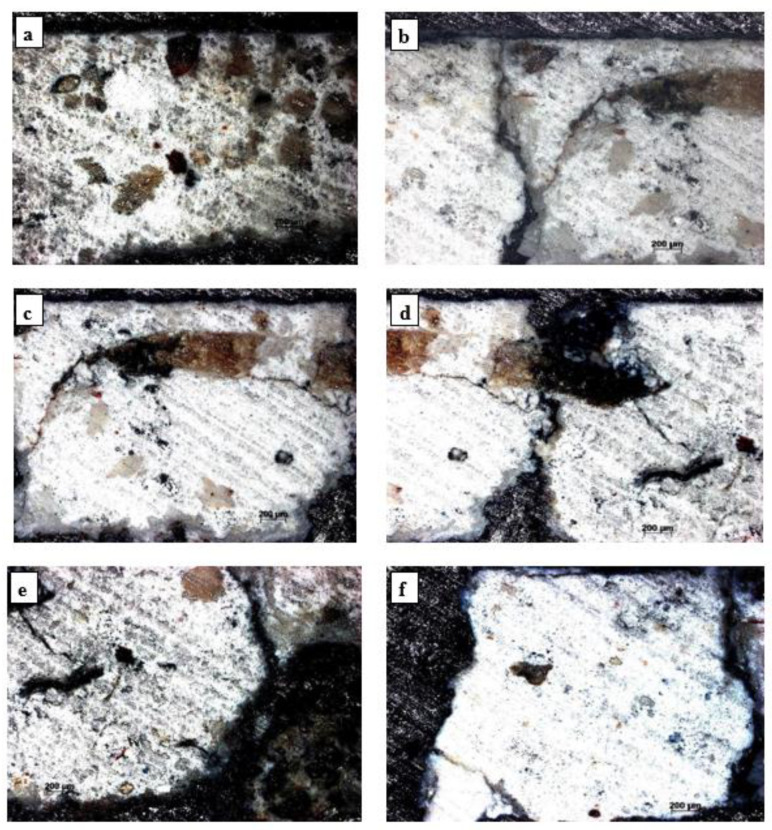
Mortar microstructure by microscopy in polarized light (OMPL): (**a**) hard inclusions in the mortar; (**b**) propagation of the crack from the top of the row and of the contraction crack in the vertical plane; (**c**,**d**) degradation by cracking; (**e**,**f**) inclusions, compactness defects.

The sample analyzed in polarized light (Figure 2) shows a pictorial layer of a dark color, very thick and of uneven thickness. Inserts, inclusion particles, pores, cracks and even voids were identified in the mortar matrix. The particles present in the mortar matrix have different shapes and sizes and have higher hardness than the mortar matrix mostly, a fact highlighted by the shading effect on the matrix (Figure 2a,c,d,f). The areas where there were straw inserts used in the preparation of the mortar matrix contain decomposition compounds with carbon from straw inserts and inclusions (Figure 2b–d). Cracks developed from the tip of the straw inserts, showing that these areas acted as stress concentrators (Figure 2b,c). Cracks appeared at the mortar insert–matrix interface that propagated into the matrix (Figure 2c,d). The cracks are long; they propagate from one boundary to another (Figure 2c–e), and the form of propagation is typical of brittle fracture (Figure 2b,d).

**Figure 3:**
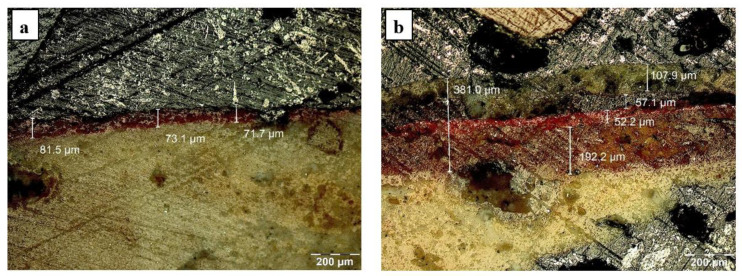
Microstructure in section of the pictorial layer: (**a**) clean layer, (**b**) layer covered with a thick crust.

The optical microscopic analysis of the painting layer revealed two situations: an area with a relatively clean outer layer, red in color, with a thickness of approximately 50 µm (Figure 3a), and an area with a thick layer, consisting of deposits, with a thickness greater than 100 µm, under which there is a layer of red pigment with a thickness of approximately 50 µm. Under the pigment layer there is a light red area, with a thickness greater than 150 µm, the intensity of the color decreases from the pigment layer towards the interior of the mortar, which shows that the pigment has diffused into the mortar (Figure 3b). The phenomenon is typical for a fresco painting on a wet support.

### 2.2. Characterization by Cathodoluminescence

For characterization by cathodoluminescence, the intensity and color of the luminescence of sedimentary particles and mortar were taken into account, in order to determine their mineralogy and to understand the behavior of these elements in conditions of excessive humidity specific to caves.

**Figure 4:**
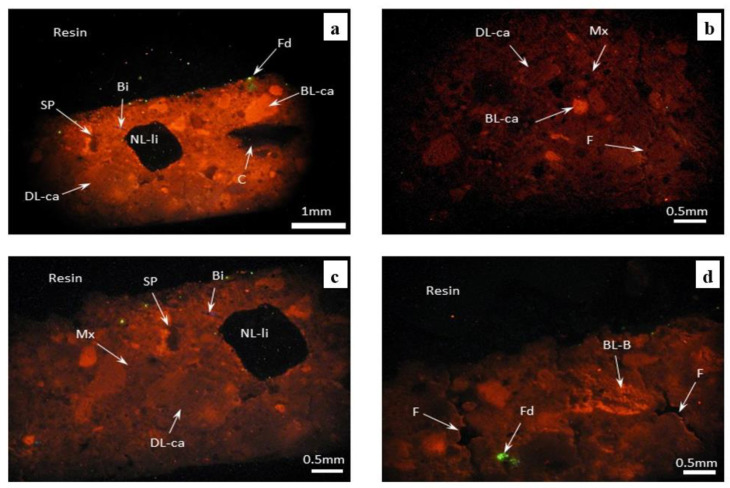
(**a**–**d**) Fragment of fresco under cathodoluminescence microscopy. BL-ca—carbonate lithoclast with bright orange luminescence; DL-ca—carbonate lithoclast with dull luminescence; NL-li—non luminescent uncertain lithoclasts; Fd—feldspar grain with green bright luminescence; Bi—biotite with dull blue luminescence; BL-B—bioclasts wih bright orange luminescence; C—carbonized plant fragments; Mx—dull luminescent matrix; SP—secondary porosity resulting from selective dissolution; F—irregular fissures.

Characterization by cathodoluminescence of fresco fragment (Figure 4) revealed small fragments of carbonate and non-carbonate sedimentary grains (sand) embedded in a relatively homogeneous matrix. The sedimentary particles (fine sand) are represented by small fragments of limestone, feldspar, quartz, micas (mainly biotite) and carbonate bioclasts (shell debris). The matrix is the hardened slaked lime, added to the mixture at the point of the mortar preparation process.

The granulometry of the particles involved is different, with variable sizes from 62 to 1000 µm, and they have subrounded contours and very poor sorting. Non-carbonate grains from the analyzed composition are represented by feldspars and micas (mainly biotite). Both are found in low concentrations. The carbonate grains and shell debris show different colors and luminescence, with some having bright orange luminescence and others showing dull luminescence. The feldspars show bright green luminescence and biotite dull blue-violet luminescence. Sometimes, secondary transformations can be observed on the surface of the feldspars.

The strong luminescence is due to the presence of some activator mineral impurities, such as Mn^2+^, which easily substitute the elements Ca^2+^ or Mg^2+^ existing in the original crystalline network of the carbonate particles [15,16]. Dull luminescence suggests a precipitation environment where activator elements (Mn^2+^) coexist with inhibitory elements (Fe^2+^) [16,19].

The mortar which binds the sand fragments has a dull, reddish-brown luminescence, which suggests that the raw material used to obtain it comes from a dolomitic source, possibly dolomitic lime. The dull luminescence of the mortar is the result of its preparation under exogenous conditions, oxygen being an inhibitory factor of bright luminescence. The non-luminescent particles of an organic nature (straw), used in the mortar recipe were also identified.

In the analyzed mass, an incipient secondary porosity is observed, with the appearance of small and irregular pores in low concentration. The secondary porosity resulting from selective dissolution was generated by the interaction of both the sedimentary particles and mortar with meteoric waters, which have a slightly acidic character (pH = 5.5–6.5), and allow the fractional and selective dissolution of the particles involved [20]. Fine, irregular cracks also be observed, with approximately perpendicular development on the external surface of the fresco, due to the seasonal repetitive processes of drying and wetting.

The cathodoluminescence analysis highlighted the degradation process of the mortar layer throughout its thickness, with many pores and irregular cracks, which explains its friable character and low adhesion to the tiles, as well as the easy detachment of the fresco from the wall on large portions.

### 2.3. Atomic Force Microscopy

Atomic force microscopy is used in the analysis of composite materials and paints to highlight the surface profile and to characterize the surface condition of these materials [21,22]. Atomic force microscopy (AFM) was performed on an Easy Scan II from NanoSurf using a PPP-CTR10 Silicon tip in contact mode. The main monitored parameters were the average roughness (Ra) and the average squared roughness (Rq) characteristic of a line on the investigated surface or Sa and Sq the corresponding parameters for the entire analyzed surface [22]. The average roughness (Ra) represents the average value of the individual heights (surface roughness) and the depth of the arithmetic mean elevation of the profile. The root mean square roughness (Rq) represents the square root of the sum of the squares of the individual heights and depths from the mean line of the investigated line.

**Figure 5:**
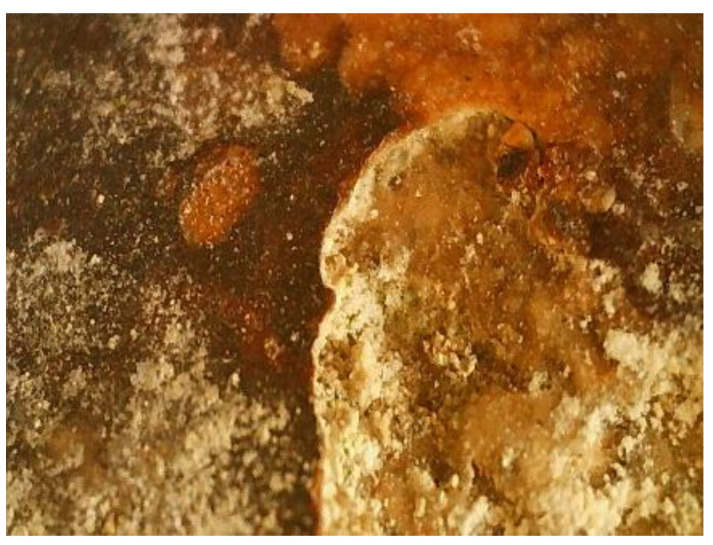
The mortar material present under the black pictorial layer, analyzed by atomic force microscopy.

The purpose of the experiments was to determine the average surface roughness of the materials, their 2D and 3D appearance and to identify the use within the materials of a unitary homogeneous aggregate made of a single material or a multi-component one. The appearance of the surface of composite materials covered with a pictorial layer was also observed. The construction material and mortar, under the black painting layer that was investigated by atomic force microscopy is shown in Figure 5.

**Figure 6:**
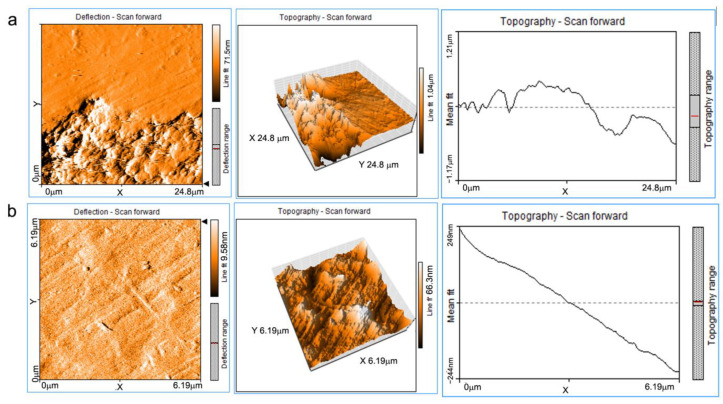
AFM images of the surface of the material of sample N: (**a**) on an analysis surface of 25 × 25 μm^2^ and (**b**) 6 × 6 μm^2^.

Figure 6 shows two surfaces at 25 × 25 and 6 × 6 μm2, respectively, on the surface of the material from the side not exposed to light. Figure 6a shows that the sample is made up of at least two types of materials with very different levels of roughness: Ra = 27.09 nm and Rq = 31.18 nm; and for the second area: Ra = 330 nm and Rq = 409.6 nm.

For the entire surface, the Sa and Sq parameters have values of 135.17 and 215.6 nm, respectively.

For the second surface (Figure 6b), the main parameters of the surface condition are: Sa = 8.09 nm and Sq = 10.44 nm. At the nano metric level, different materials or different structural constituents are not observed, they appear only at the micrometric level. At micrometric scale exists an intimate contact between the component materials of the aggregate without a clear boundary between them, with a good cohesion that supports the good general material properties.

### 2.4. The Morphological and Chemical Elemental Analysis

The morphological and chemical elemental analysis by SEM-EDS was performed for both the mortar support layer and for the upper pictorial layer [11,12,23,26,27].

**Figure 7:**
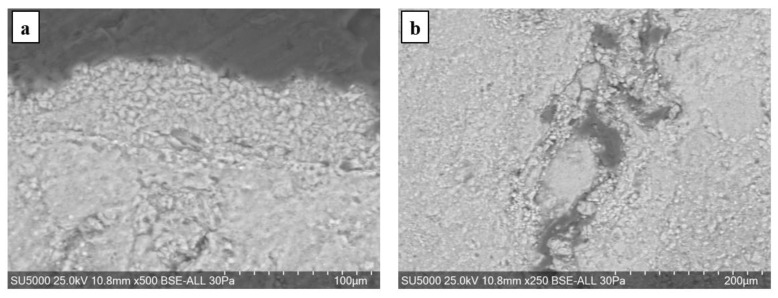
The microstructure of the fresco sample in cross-section: (**a**) the intonaco layer (×500 mag.); (**b**) advanced degradation zone (×250 mag.).

**Figure 8:**
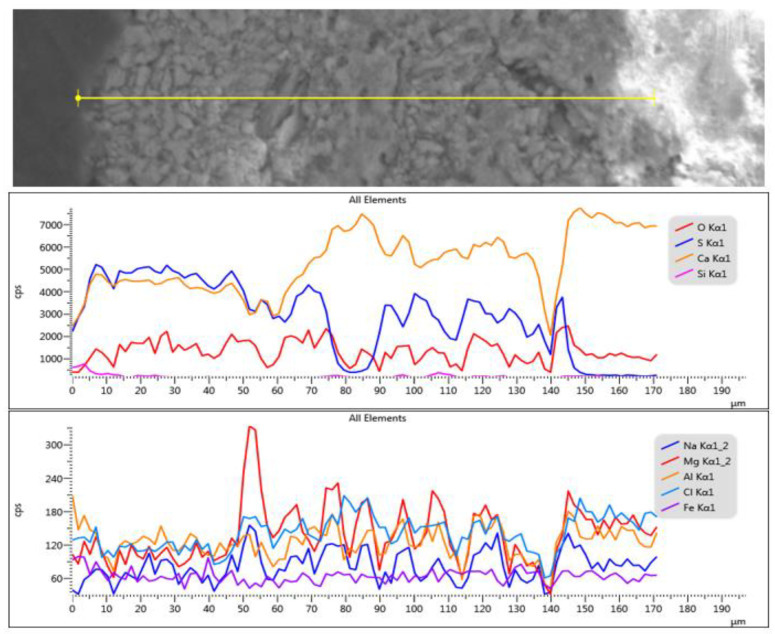
SEM-EDS line-scan analysis on the thickness of the mortar layer.

The SEM analysis of the fresco element shows that the pictorial layer is very thin, barely visible. The intonaco layer shows important degradations and a low compactness, towards a granular structure (Figure 7 and Figure 8). The granular structure, with uneven areas, does not allow a delimitation of the intonaco and arriccio layers. Inside the mortar layer, areas of advanced degradation were highlighted, with large compaction defects (Figure 7b).

**Figure 9:**
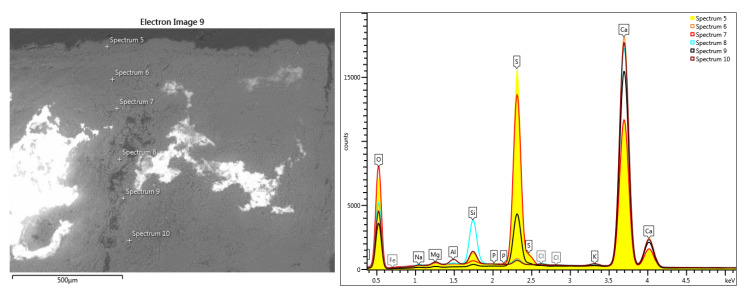
Superimposed spectra regarding the evolution of the composition on the depth of the fresco: superficial layer with advanced pollution and Fe traces; the specific elements of the mortar composition.

The line-scan analysis, on the thickness of the mortar layer, is presented in Figure 8. From the point of view of the composition, the presence of magnesium and calcium is noted throughout the thickness of the mortar. The superimposed spectra of the composition determined at different points located from the surface to the interior, in an area without pronounced degradation of the mortar layer (Figure 9), show high concentrations of sulfur near the surface (spectra 5,6), and a decrease of sulfur concentration at the surface of the layer towards its interior (spectra 9,10).

The variation of the chemical composition on the thickness of the pictorial layer sample towards the interior of the mortar, without an advanced internal degradation, shows us the composition of the pictorial layer of the sample surface and the evolution of the mortar composition (Figure 9 and Table 1).

**Figure 10:**
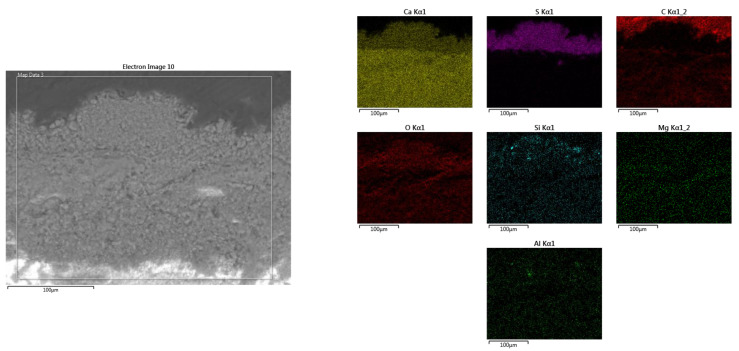
SEM-EDS mapping of the elemental composition in an area where the mortar layer does not show advanced degradation inside, with high sulfur content on the surface.

EDS-mapping drawn in the area without loosening on the thickness of the fresco layer (Figure 10) shows the presence of sulfur on the surface, which can be explained by the infiltration of meteoric water, which causes degradation of the fresco.

**Figure 11:**
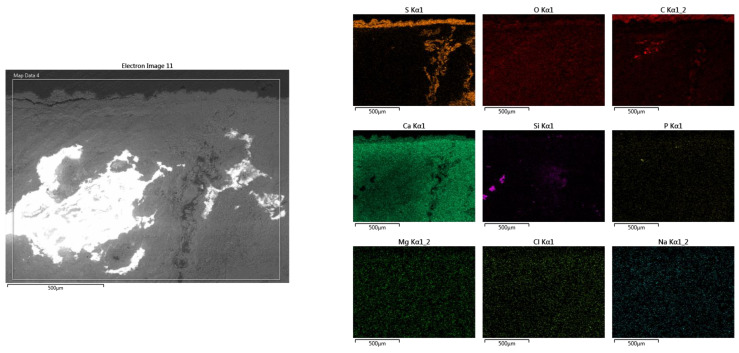
SEM-EDS mapping of the elemental composition in the mortar degradation zone through the infiltration of meteoric waters, with high sulfur content on the surface and in the advanced degradation zone.

For the area of the mortar layer showing advanced internal degradation (Figure 7b), the elementary SEM-EDS analysis highlighted the correlation between the high sulfur content and the area of advanced degradation of the mortar layer (Figure 11).

**Figure 12:**
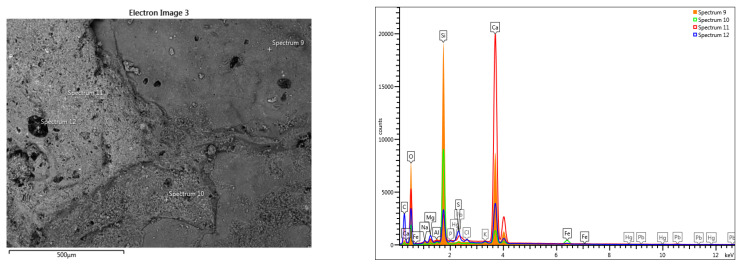
SEM-EDS scan on the surface of the painting layer: Fe, Hg, Pb.

**Figure 13:**
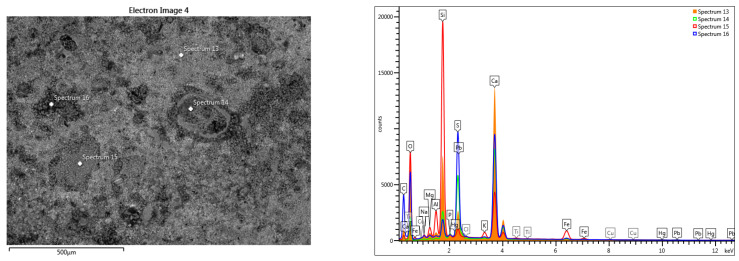
SEM-EDS scan on the surface of the painting layer: Fe, Hg, Pb and Ti traces.

**Figure 14:**
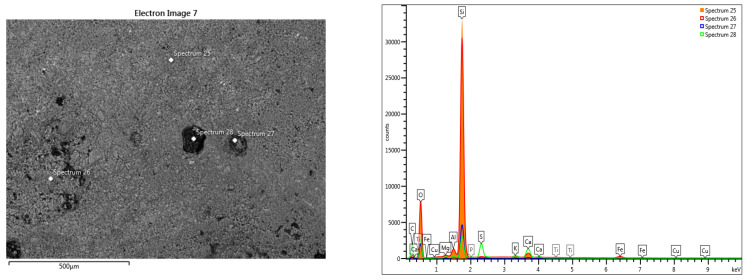
SEM-EDS scan on the surface of the painting layer: Fe, Ti and Cu traces.

SEM analysis of the surface highlights the pictorial layer with unevenness resulting from biodegradation and impurities [10] (Figure 12, Figure 13 and Figure 14).

The elemental chemical composition determined in the points in Figure 12 and Figure 13 shows the presence of iron, mercury and lead. For the points in Figure 14, the pictorial layer shows iron (Table 2).

The elemental chemical composition determined the presence of iron and mercury in the section through the pictorial layer (Table 3). Traces of titanium and copper were also identified in the analyzed pictorial layers. Considering the determined chemical composition, it can be estimated that the pictorial layer is formed by a mixture of cinnabar (HgS) and iron red ochre (hematite Fe_2_O_3_) pigments. The presence of lead can be associated with the use of a third pigment: lead red or Saturn red (PbO_2_.2PbO). The use of a mixture of pigments is frequent in the fresco technique.

**Table 3:** Elemental chemical compositions above the paint layer, determined in the points shown in Figure 15.

Spectrum Label	Concentration in wt%—Figure 15 Point Scans														
	O	Na	Mg	Al	Si	P	S	Cl	K	Ca	Ti	Mn	Fe	Hg	Total
17	4.41	-	-	0.19	0.72	-	15.53	-	-	2.02	-	-	0.19	76.94	100.00
18	52.47	1.01	4.74	2.79	14.63	0.13	0.37	0.31	0.29	21.40	0.14	0.11	1.61	-	100.00
19	49.41	0.44	1.70	0.21	4.59	0.05	0.51	0.24	0.07	42.54	-	-	0.25	-	100.00

The presence of sodium, associated with Ca, Al, Si and S, requires the use of sodium aluminosilicate with sulfur or ultramarine blue (Na, Ca)_8_ (AlSiO_4_)_6_ (SO_4_, S, Cl)_2_ [30]. the previous research did not ignore the presence of a blue pigment, so this possibility must be checked.

### 2.5. Qualitative Phase Analysis by X-ray Diffraction

The qualitative phase analysis [25,28,29] was carried out using the PDXL2 program (Rigaku) and the PDF4+ 2022 database (International Center for Diffraction Data).

**Figure 16:**
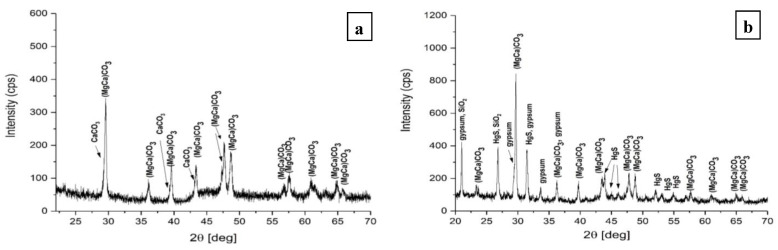
Qualitative phase analysis by X-ray diffraction of the fresco element with low adhesion to the sandstone wall: (**a**) mortar; (**b**) pictorial layer.

The XRD qualitative phase analysis (Figure 16) highlighted the presence of the following polycrystalline phases:

- For the mortar support, Figure 5a: calcium carbonate (CaCO_3_) and calcium and magnesium carbonate ((Mg_0.03Ca0_._97_)O_3_;
- For the pictorial layer, Figure 5b: cinnabar (α-HgS), gypsum (CaSO_4_(H_2_O)_2_), α-quartz (α-SiO_2_) and calcium and magnesium carbonate (Mg_0.064_Ca_0.936_ CO_3_).

The gypsum comes from the infiltration water that carries it through the sandstone to the fresco; the waters that infiltrate the sandstone rock pass through clay-limestone rocks that contain gypsum [20]. Plaster can be found in the compactness defects of the support mortar, where it recrystallizes, or on the surface of the fresco in the pictorial layer. It has an important role in the degradation of the fresco. This type of degradation is marked by the presence of sulfur and is linked to defects in the sandstone walls that allow the transport of a large volume of water.

## 3. Conclusions

The paper presents an advanced characterization of a portion of the medieval fresco from the Corbii de Piatră Hermitage Church. The element of the fresco was taken from the southern wall of the church, at 1.70 m height from the floor level, the area where the mortar became friable, and the fresco detached in large portions.

The fresco as a whole was characterized, including the mortar support and the pictorial red layer.

The mortar matrix is degraded, the structure has a granular appearance and the thickness of the intonacco layer cannot be defined and measured.

The matrix contains decomposition compounds with carbon from straw inserts and inclusions in different shapes, sizes and hardness—mostly sand.

The compactness defects in the form of pores come from both the execution of the fresco, and the action of the weather-any infiltrated water that created a connected porosity. Internal cracks start from the top of their straw inserts, from the mortar-insert interface and from defects. They have grown to great lengths and have a fragile appearance.

The cracks in the surface of the fresco are developed perpendicular to the surface and are due to repeated wetting/drying processes.

The processes of internal degradation of the mortar layer through the action of meteoric water are correlated with the presence of sulfur in the strongly degraded area.

The red pictorial layer can be observed with the naked eye only in areas where it is relatively clean. It is mostly covered by a layer of deposits with a great thickness.

In the upper part, areas of protective carbonate were identified, specific to the carbonation process during the drying of the fresco, along with crusts and efflorescent compounds formed over time.

Under the layer of red pigment, there is a large area of pigment diffusion in the mortar, a phenomenon specific to the execution of the fresco on a wet wall.

In the upper part, areas of protective carbonate were identified, specific to the carbonation process during the drying of the fresco.

The red pigments identified in the pictorial layer are red iron ochre (Fe_2_O_3_), cinnabar (HgS), and min of Pb (Pb_3_O_4_). Mixtures of natural and synthetic, native and imported pigments were used to create the shade and intensity of the color. A complementary study is provided to verify the presence of other pigments.

The decrease in adhesion of the fresco to the wall is due to the degradation of the mortar matrix, mainly through the action of meteoric infiltration water.

Considering the advanced degradation of the support mortar produced by the penetration of the meteoric filtration water throughout its thickness, and the fact that, due to the location of the church, the process of water infiltration through the sandstone wall cannot be eliminated, we consider that the use of restoration mortars similar to those used in the execution of the fresco cannot solve the stability of the fresco on the walls.

Solutions for the restoration of the fresco should take into account the possibility of using some drainage mortars for the hedgehog layer, over which the intonaco layer for the fresco is applied [31]. In order to increase the properties of the mortar matrix, additives (mineral oil or sheep tallow) can be used that would increase its density and mechanical characteristics.

## Figures and Tables

**Figure 15:**
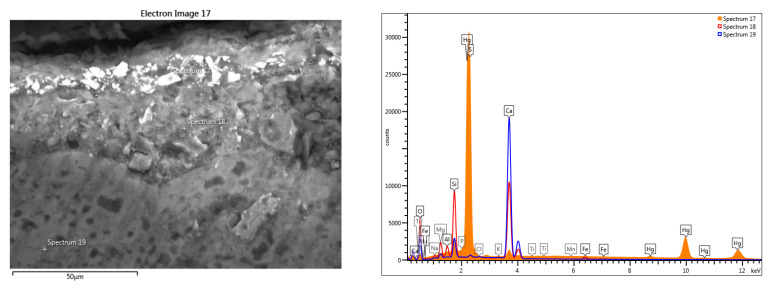
SEM-EDS of the red pictorial layer: cross-section scan through the fresco fragment (Hg, Fe).

**Table 1:** Elemental chemical compositions above the paint layer, determined in the points shown in Figure 9.

Spectrum Label	Concentration in wt% Figure 9. Point Scans					
	5	6	7	8	9	10
O	58.55	58.39	62.02	58.42	54.16	57.65
F	-	-	-	-	-	-
Na	0.08	0.33	0.10	0.40	0.20	0.33
Mg	0.14	0.53	0.31	0.54	0.25	0.67
Al	0.11	0.04	0.04	0.12	-	0.75
Si	1.09	1.45	0.28	5.11	0.29	1.65
P	0.02	0.08	-	0.04	-	-
S	18.42	0.73	15.95	0.55	6.88	0.52
Cl		0.13		0.15	0.18	0.15
K	0.11	0.21	0.08	0.21	0.15	0.30
Ca	21.47	38.12	21.22	34.46	37.89	37.73
Ti	-	-	-	-	-	-
Fe	-	-	-	-	-	0.25
Hg	-	-	-	-	-	-
Pb	-	-	-	-	-	-
Total	100.00	100.00	100.00	100.00	100.00	100.00

**Table 2:** Elemental chemical compositions above the paint layer, determined in the points shown in Figure 12, Figure 13 and Figure 14.

Spectrum Label	Concentration in wt% Figure 12 Point Scans				Concentration in wt% Figure 13 Point Scans				Concentration in wt% Figure 14 Point Scans			
	9	10	11	12	13	14	15	16	25	26	27	28
O	56.55	42.65	57.03	57.04	58.16	43.67	50.43	58.58	48.35	50.17	56.24	59.67
Na	0.30	-	0.43	1.74	0.59	0.31	0.66	0.63	-	-	-	-
Mg	0.76	0.81	0.50	3.52	0.61	0.42	1.77	0.58	0.39	0.36	0.39	1.08
Al	0.27	0.74	0.11	0.38	0.46	0.21	3.63	0.19	1.25	1.47	1.56	0.85
Si	24.94	39.34	3.60	10.21	9.33	5.95	27.59	2.40	46.62	44.63	36.91	14.46
P	-	-	-	0.58	0.17	1.21	0.70	0.29	-	-	-	0.35
S	0.24	0.75	0.23	3.23	2.78	15.50	1.33	15.71	0.11	0.20	0.31	11.53
Cl	-	-	0.11	1.32	-	0.24	-	-	-	-	-	-
K	-	0.79	-	0.60	0.16	-	0.99	0.22	0.53	0.42	0.44	1.93
Ca	16.55	8.25	36.23	17.07	23.86	29.39	8.36	20.63	1.14	1.41	1.21	8.76
Ti	-	-	-	-	-	-	0.14	-	-	0.09	-	-
Fe	0.39	6.67	-	0.88	0.91	0.76	4.40	0.78	1.35	1.25	2.04	0.92
Cu	-	-	-	-	-	0.22	-	-	0.26	-	0.91	0.44
Hg	-	-	-	2.03	0.67	2.12	-	-	-	-	-	-
Pb	-	-	1.76	1.41	2.30	-	-	-	-	-	-	-
Total	100.00	100.00	100.00	100.00	100.00	100.00	100.00	100.00	100.00	100.00	100.00	100.00

## Data Availability

All data results are present in the present article.
